# *ZmRAD17* Is Required for Accurate Double-Strand Break Repair During Maize Male Meiosis

**DOI:** 10.3389/fpls.2021.626528

**Published:** 2021-02-26

**Authors:** Ting Zhang, Ju-Li Jing, Lei Liu, Yan He

**Affiliations:** ^1^Ministry of Education Key Laboratory of Crop Heterosis and Utilization, National Maize Improvement Center of China, College of Agronomy and Biotechnology, China Agricultural University, Beijing, China; ^2^Beijing Key Lab of Plant Resource Research and Development, Beijing Technology and Business University, Beijing, China

**Keywords:** maize, meiosis, DSB, HR, RAD17

## Abstract

RAD17, a replication factor C (RFC)-like DNA damage sensor protein, is involved in DNA checkpoint control and required for both meiosis and mitosis in yeast and mammals. In plant, the meiotic function of *RAD17* was only reported in rice so far. Here, we identified and characterized the *RAD17* homolog in maize. The *Zmrad17* mutants exhibited normal vegetative growth but male was partially sterile. In *Zmrad17* pollen mother cells, non-homologous chromosome entanglement and chromosome fragmentation were frequently observed. Immunofluorescence analysis manifested that DSB formation occurred as normal and the loading pattern of RAD51 signals was similar to wild-type at the early stage of prophase I in the mutants. The localization of the axial element ASY1 was normal, while the assembly of the central element ZYP1 was severely disrupted in *Zmrad17* meiocytes. Surprisingly, no obvious defect in female sterility was observed in *Zmrad17* mutants. Taken together, our results suggest that *ZmRAD17* is involved in DSB repair likely by promoting synaptonemal complex assembly in maize male meiosis. These phenomena highlight a high extent of divergence from its counterpart in rice, indicating that the *RAD17* dysfunction can result in a drastic dissimilarity in meiotic outcome in different plant species.

## Introduction

In eukaryotes, meiosis is a key biological process for reproduction with one round of DNA replication followed by two successive cell divisions (meiosis I and II) to halve chromosome number (de Massy, 2013). During meiosis I, homologous pairing and synapsis promote crossover (CO) formation, guaranteeing the accurate segregation of homologous chromosomes (Mercier et al., 2015). Thus, this division is also called as reductional division (Ma, 2006). Subsequently, meiosis II (also called equational division) leads to sister chromatids separation (Zickler and Kleckner, 1999; Ma, 2006). The biological significances of meiosis are to maintain genome stability and boost the genetic diversity between offspring through homologous recombination (HR) (Zickler and Kleckner, 2015).

HR is initiated by the programmed formation of DNA double-strand breaks (DSBs), which are catalyzed by a topoisomerase-like protein SPO11 and several accessory proteins (Keeney et al., 1997; Lam and Keeney, 2015). DSB sites are further resected by a protein complex known as MRX/N (Mre11-Rad50-Xrs2/Nbs1) and Sae2/Com1/CtIP/Ctp1 (Lamarche et al., 2010; Wang et al., 2018), generating replication protein A (RPA)-coated single-stranded DNA (ssDNA) overhangs (Mimitou and Symington, 2009). Then, RPA is replaced by the RecA recombinases RAD51 and DMC1 forming nucleoprotein filaments and promoting homology search and single strand invasion to produce recombination intermediates called as the displacement (D)-loop (Hunter and Kleckner, 2001; Cloud et al., 2012). Ultimately, the extended D-Loop gives rise to double Holliday Junction (dHJ), which is resolved into a minority of COs and large number of NCOs (Youds and Boulton, 2011; Pyatnitskaya et al., 2019).

RAD17, a replication factor C (RFC)-like protein, is required for responses to DNA damage, replication stress and DSB repair (Shinohara et al., 2003; Wang et al., 2003, 2006b; Budzowska et al., 2004). The mechanism of RAD17 has been well illustrated in several species, such as yeast and human cells. In general, RAD17 acts as the checkpoint clamp loader to recruit the 9-1-1 complex (RAD9/HUS1/RAD1) onto DSB sites to promote interhomolog recombination and crossover formation (Burtelow et al., 2001; Zou et al., 2001; Griffith et al., 2002; Parrilla-Castellar et al., 2004; Majka et al., 2004; Navadgi-Patil and Burgers, 2009; Liu, 2019). In human, RAD17 facilitates the MRE11-RAD50-NBS1 complex loading and regulates the response to DNA damage (Wang et al., 2014). RAD17 functions relatively comprehensive in yeast. In budding yeast, Rad24 (the homolog of RAD17) was not only necessary for Ddc1/Mec3/Rad17 (the homolog of Rad9/Hus1/Rad1, respectively) loading onto DSB sites, but also required for meiotic prophase arrest in *dmc1* mutant background (Lydall et al., 1996; Majka and Burgers, 2005).

In plant, the mutation in *AtRAD17* led to hypersensitivity to the DNA-damaging agent treatment, whereas mutant plants were fully fertile, suggesting that the *RAD17* may not play an important role in Arabidopsis meiosis (Heitzeberg et al., 2004). In contrast, the disruption in *OsRAD17* resulted in aberrant associations between non-homologous chromosomes, leading to massive chromosome entanglements and fragmentations, indicating that the *OsRAD17* is essential for meiotic DSB repair in rice (Hu et al., 2018). The marked dissimilarity of meiotic outcomes caused by the defective *RAD17* raises an intriguing question that whether the role of *RAD17* in meiosis is conserved across plant kingdom. In this study, we characterized the maize *ZmRAD17* using a reverse genetic approach. Our results demonstrate that *ZmRAD17* is required for accurate DSB repair only in male meiosis. We also show that the meiotic abnormalities in *Zmrad17* exhibit multifaced differences from its counterpart in rice, implying that although the roles of *RAD17* in DSB repair seem to be fundamentally conserved at least in grass species, the exactly operative manner of *RAD17* may vary in different plant organisms.

## Materials and Methods

### Plant Materials

We obtained two *Zmrad17* mutants from the Maize EMS induced Mutant Database (MEMD)^1^ (Lu et al., 2018). All plants were grown in field during the growing season or greenhouse under normal growth conditions. Primer sequences used in genotyping were listed in Supplementary Table S1.

### Pollen Viability

Pollen grains were dissected out of fresh anthers during pollination stage and viability was assessed by 1% I_2_-KI staining. Images of stained pollen grains were taken using a Leica EZ4 HD stereo microscope equipped with a Leica DM2000 LED illumination system (Leica, Solms, Germany).

### Rapid Amplification of cDNA Ends (RACE) and Reverse Transcription Quantitative PCR (RT-qPCR) Analysis

Total mRNA was isolated from root, stem, leaf, developing meiotic ear (1-2cm in length), immature tassel, developing embryo and endosperm (16 days after pollination) of B73 plants with TRIzol (TIANGEN). cDNA synthesis was performed by TaKaRa kits according to manufacturer’s instructions. The entire cDNA was cloned by RACE using the SMART RACE cDNA amplification kit (Clontech). RT-qPCR analysis was performed using the CFX Connect Real-Time PCR System (BIO-RAD). Primer sequences used in RT-qPCR were listed in Supplementary Table S1.

### Preparation of Meiotic Chromosome Spreads

Immature tassels were fixed for 24 h in Carnoy’s solution (ethanol: acetic acid = 3:1, v/v). Then, tassels were stored in 70% ethanol at 4°C. Anthers at meiotic stages were squashed in 45% (v/v) acetic acid solution. Slides with chromosomes were frozen in liquid nitrogen and then cover slips were removed immediately. The slides were dehydrated through an ethanol series (70/90/100%) for 5 min each once. Dried slides were stained with 4′,6-diamidino-2-phenylindole (DAPI) in an antifade solution (Vector). Images were captured using a Ci-S-FL microscope (Nikon, Tokyo) equipped with a DS-Qi2 Microscope Camera system.

### Florescence *in situ* Hybridization (FISH)

The FISH analysis was performed according to protocols described previously (Richards and Ausube, 1988; Li and Arumuganathan, 2001; Wang et al., 2006a; Han et al., 2007; Cheng, 20136a). Two repetitive DNA elements, 5S rDNA repeats (pTa794) and the telomere-specific repeats (pAtT4), were used as probes (Richards and Ausube, 1988). Probes were labeled with digoxigenin by nick translation mix (Roche) and detected with anti-digoxigenin antibody (Vector). Chromosome images were captured under a Ci-S-FL fluorescence microscope (Nikon) equipped with a DS-Qi2 microscopy camera (Nikon, Tokyo, Japan).

### Immunofluorescence Assay

Young anthers during meiotic stages were fixed in 4% (w/v) paraformaldehyde for 30 min at room temperature and stored in 1x Buffer A at 4°C. Immunofluorescence was performed as previously described (Pawlowski et al., 2003; Cheng, 2013). The primary antibodies against ASY1, ZYP1, and γH2AX were prepared as described previously (Jing et al., 2019). Antibody against RAD51 was a gift from Wojtek Pawlowski’s Lab at Cornell University. Fluorochrome-coupled secondary antibodies (ABclonal) were used for fluorescence detection. All primary and secondary antibodies were diluted at 1:100. Images of meiocytes were observed and captured using a Ci-S-FL microscope (Nikon) equipped with a DS-Qi2 microscopy camera (Nikon, Tokyo, Japan). The images were captured by software NIS-Elements and colored by the ImageJ software.

### Chiasma Quantification

The number of chiasmata were quantified for meiocytes at diakinesis. The rod-, ring-and “∞”-shaped bivalents were scored as one chiasma and two, three chiasmata, respectively.

## Results

### Identification of *ZmRAD17*

**FIGURE 1:**
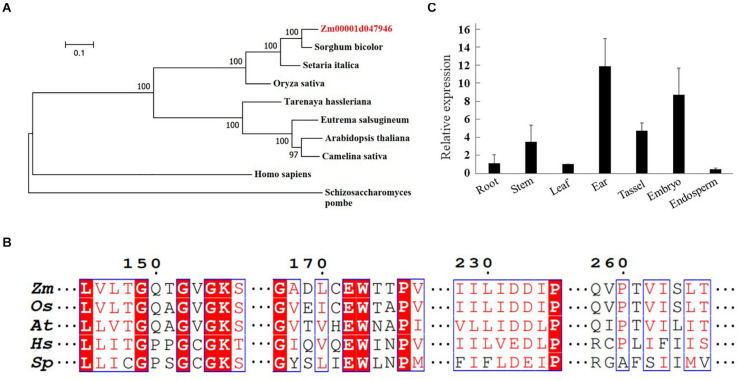
Identification of ZmRAD17. **(A)** Phylogenetic analysis of RAD 17 proteins from representative dicotyledons, monocotyledons and mammal. The neighbor-joining method was used to construct an unrooted tree. **(B)** The conserved AAA-ATPase domain of RAD 17. Zea mays (Zm); Oryza sativa (Os); Arabidopsis thaliana (At); Schizosaccharomycespombe (Sp); Homo sapiens (Hs). **(C)** Tissue-specific expression analysis of ZmRAD 17 by RT-qPCR. The relative expression was calculated from the ratio of the expression in other tissues compared with leaf after normalizing by the ZmUBQ1 (Zm00001d010159) expression. Relative expression levels were mean values of three independent experiments with standard deviation.

To identify a putative *RAD17* gene in maize, the full-length amino acid sequence of the rice *RAD17* was used as a query to search in the maize genome database^2^ by BLASTp analysis. We identified only one candidate gene (*Zm00001d047946*) with the highest similarity to the rice *RAD17* (*LOC_Os03g13850*). Phylogeny analyses revealed that RAD17 homologs formed two distinct clades reflecting the divergence between monocot and dicot plants (Figure 1A). In addition, the multiple sequence alignment of ZmRAD17 amino acid with its orthologs indicated that the RAD17 proteins were conserved in the primary AAA-ATPase domains (Figure 1B). We then investigated the spatio-temporal expression pattern of *ZmRAD17* using RT-qPCR analyses. The result showed that *ZmRAD17* was highly expressed in the developing tassel, ear, and embryo, but weakly expressed in root, stem, leaf and endosperm (Figure 1C).

### Characterization of *Zmrad17* Mutants

**FIGURE 2:**
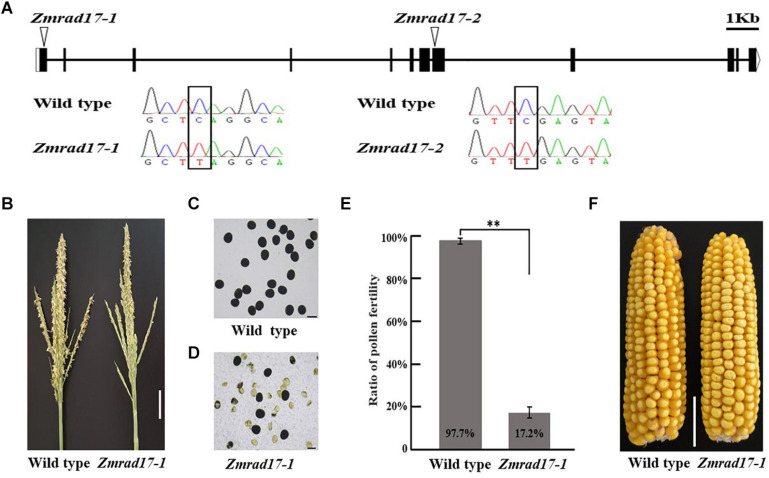
Characterization of Zmrad17 mutant. **(A)** Gene structure of ZmRAD17. Mutation sites marked with triangles. Bars indicate exons and lines represent introns. Sequence analysis detected a single nucleotide substitute C in wild type to T in Zmrad17 mutants lead to premature translation termination. Bar = lkb. **(B)** Comparison of wild type tassel and Zmrad17-1 mutant tassel. Bar = 5 cm. **(C)** Pollen grains stained with I2-KI in wild type. Three biological and three technological replicates were used. Bar = 100 μm. **(D)** Pollen grains stained with I2-KI in Zmrad17-1. Three biological and three technological replicates were used. Bar = 100 μm. **(E)** Statistics analysis of pollen fertility in wild type and Zmrad17-1. Values are means ± *SD*. Double asterisks indicates the statistical significance at *p* < 0.01 using a two-tailed Student’s *t*-test **(F)** Seed setting rate of wild type and Zmrad17-1 (homozygote) pollinated with wild type pollen. Bar = 3 cm.

The full-length cDNA sequence of *ZmRAD17* was isolated by performing rapid amplification of cDNA ends (RACE). It contains 2,089 bp with an open reading frame of 1,851bp and consists of 12 exons and 11 introns (Figure 2A). To characterize biological functions of *ZmRAD17*, two independent stop codon mutants were obtained from the EMS induced Mutant Database (MEMD) in B73 background (Lu et al., 2018). By conducting locus-specific PCR amplification followed by Sanger sequencing, we confirmed that the stop codon mutation sites are located in the first exon (named as *Zmrad17-1*) and the eighth exon (named as *Zmrad17-2*) of *ZmRAD17*, respectively (Figure 2A). Both *Zmrad17* mutants exhibited normal vegetative growth, but partially male-sterile (Figure 2B and Supplementary Figure S1A). KI-I_2_ staining displayed that unlike large, round and purple pollen grains of the wild-type (Figure 2C and Supplementary Figure S1B), a proportion of mutant pollen grains were empty, shrunken and unable to stain (Figures 2D,E and Supplementary Figures S1C,D). Surprisingly, when pollinated with pollen grains from wild-type plants, mutant ears exhibited a similar extent of seed setting (Figure 2F and Supplementary Figure S1E). These results indicate that the dysfunction of *ZmRAD17* causes effects on male reproductive development, but not on female.

### Abnormal Meiotic Chromosome Behaviors in *Zmrad17* Mutants

**FIGURE 3:**
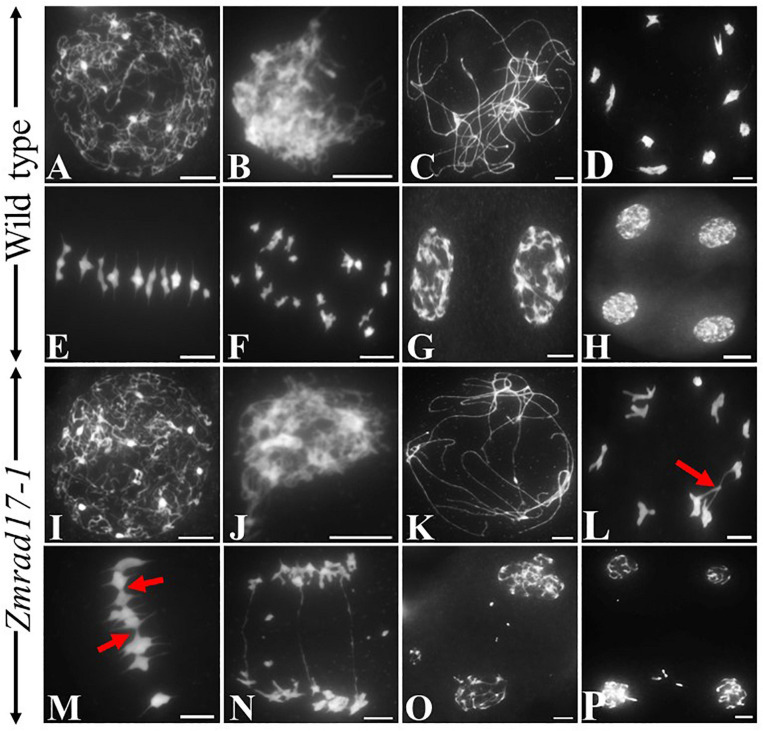
The abnormal chromosome behaviors in Zmrad17-1 meiocytes. **(A–H)** Meiosis in the wild type. (I-P) Meiosis in the Zmrad17-1 mutant. **(A,I)** Leptotene; **(B,J)** Zygotene; **(C,K)** Pachytene; **(D,L)** Diakinesis; **(E,M)** Metaphase I; **(F,N)** Anaphase I; **(G,O)** Telophase I; **(H,P)** Tetrad. The red arrows pointed out the association between non-homologous chromosomes. Bars = 10 μm.

To explore whether pollen abortion is resulted from the defect in male meiosis, chromosome behaviors were investigated in both wild-type and *Zmrad17* meiocytes at different stages by staining chromosome spreads with 4′,6-diamidino-2-phenylindole (DAPI). In the wild-type, chromosomes begun to condense and became visible as thin threads structures at leptotene (Figure 3A). Then, homologous chromosomes came close to each other and started to pair and synapsis at zygotene (Figure 3B). During pachytene, chromosomes were fully synapsed to form thick threads (Figure 3C). With chromosomes further condensed, 10 short, rod-like bivalents appeared to scatter in the nucleus at diakinesis (Figure 3D). Once entry into metaphase I, ten bivalents aligned on the equatorial plate in an orderly manner (Figure 3E). At anaphase I, homologous chromosomes separated equally and migrated toward the opposite poles (Figure 3F) forming dyad (Figure 3G). After the second meiotic division, the sister chromatids segregated and ultimately produced tetrad (Figure 3H).

In both of *Zmrad17* mutant meiocytes, chromosome behaviors were indistinguishable from the wild-type from leptotene to zygotene (Figures 3I,J and Supplementary Figures S2A,B). However, meiotic abnormalities started to be constantly observed at pachytene, showing abnormal chromosome associations between non-homologous chromosomes (Figure 3K and Supplementary Figure S2C). At diakinesis, although ten bivalents formed, aberrant bridges among bivalents were frequently observed in *Zmrad17* meiocytes (Figure 3L, *n* = 37; Supplementary Figure S2D). Despite all bivalents could be aligned on the equatorial plate during metaphase I, *Zmrad17* meiocytes exhibited abnormal bivalent aggregation (Figure 3M and Supplementary Figure S2E). At anaphase I, homologous chromosomes separated with obvious chromosome bridge and chromosome fragmentation (Figure 3N and Supplementary Figure S2F). Chromosome fragments were lagged and scattered randomly within the nucleus at telophase I (Figure 3O and Supplementary Figure S2G). The second meiotic division subsequently underwent and tetrad with micronuclei were formed (Figure 3P and Supplementary Figure S3H). These results suggest that the abnormal chromosome behaviors are responsible for the male sterility of *Zmrad17* mutants. Since *Zmrad17-1* and *Zmrad17-2* exhibited the same defect in the meiotic chromosome behaviors, all subsequent analyses were conducted using *Zmrad17-1* mutant as a representative of the *Zmrad17* dysfunction.

### *ZmRAD17* Is Not Required for DSB and CO Formation

**FIGURE 4:**
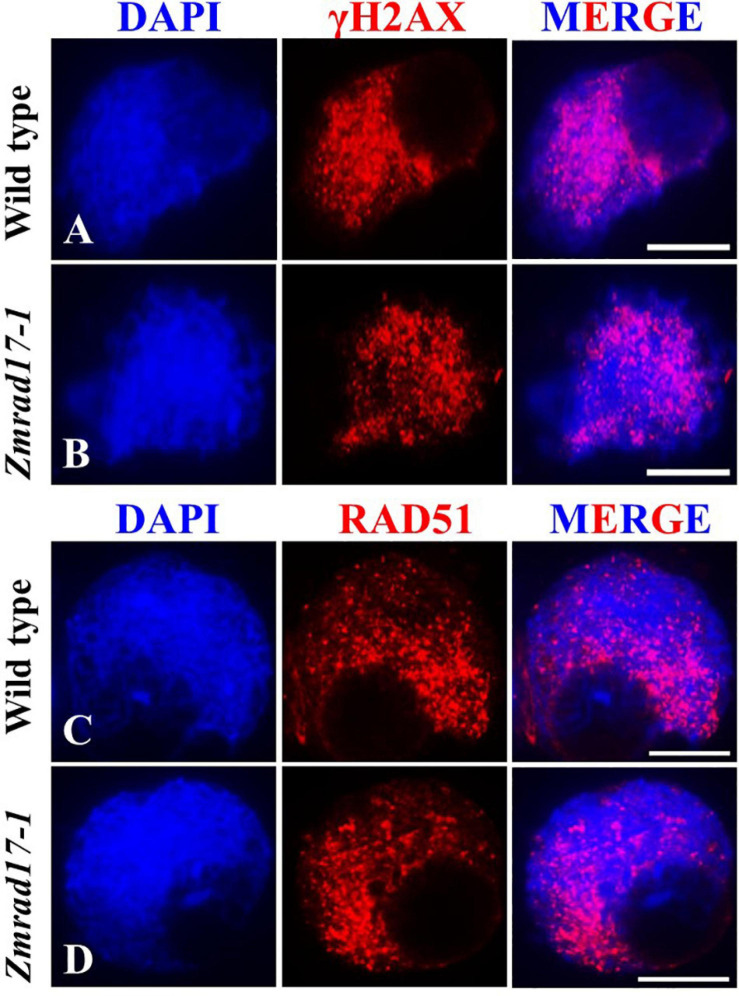
ZmRAD17 is not required for DSB formation. **(A,B)** γH2AX foci in wild type **(A)** and Zmradl 7-1 meiocytes **(B)**. **(C,D)** RAD51 foci in wild type **(C)** and Zmrad17-1 meiocytes **(D)**. DAPI staining was used to indicate the chromosomes. Bars = 10 um.

To evaluate whether DSB formation is defective in *Zmrad17* mutant, we performed immunostaining with antibodies against γH2AX and RAD51. γH2AX is a specific histone variant accumulating at damaged sites to promote DSB repair (Hunter et al., 2001; Dickey et al., 2009). Therefore, γH2AX is routinely used as a cytogenetic marker to detect the presence of DSB (Valdiglesias et al., 2013; Geric et al., 2014; Turinetto and Giachino, 2015). Our analysis revealed a substantial amount of dot-like γH2AX signals appeared in both wild-type (Figure 4A, n = 13) and mutant meiocytes at zygotene (Figure 4B, *n* = 16), suggesting that *ZmRAD17* is dispensable for DSB formation. The loading of RAD51 on chromosomes serves as an important marker to monitor HR-mediated DSB repair in many different organisms (Pawlowski et al., 2003). Constantly, we did not observe marked difference in the localization of RAD51 signals between wild-type (Figure 4C, *n* = 24) and *Zmrad17-1* meiocytes at zygotene (Figure 4D, *n* = 35), suggesting that *ZmRAD17* is not crucial for HR initiation. Moreover, the number of chiasmata were counted in both wild type and mutant meiocytes at diakinesis stage using a method described previously (Moran et al., 2001). We found that although aberrant associations among bivalents occurred in *Zmrad17-1* meiocytes, the number of chiasmata (Supplementary Figure S3) seemed comparable between wild type (17.08 ± 1.93, *n* = 24) and mutant (17.11 ± 2.10, *n* = 19), implying that *ZmRAD17* is not critical for CO formation.

### *ZmRAD17* Is Dispensable for Telomere Bouquet Clustering and Homologous Pairing

**FIGURE 5:**
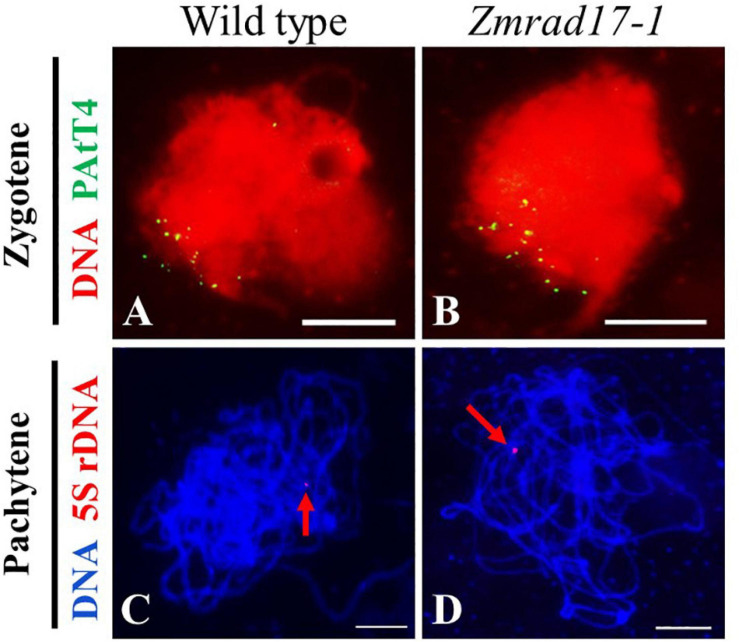
ZmRAD17 is not required for telomere bouquet formation and homologous pairing. **(A,B)** FISH with the telomere-specific pAtT4 probe in the wild type **(A)**, Zmrad17-1 **(B)** meiocytes at zygotene. **(C,D)** FISH with the 5S rDNA the wild type **(C)** and Zmrad17-1 **(D)** meiocytes at pachytene. The red arrows pointed out the 5S rDNA signal. Bars = 10 um.

Telomere bouquet clustering occurs specifically at early zygotene and is thought to be essential for homologous pairing and synapsis (Bass et al., 1997; Harper et al., 2004). To test whether telomere bouquet formation is affected in *Zmrad17-1*, we conducted FISH using a telomere specific probe (pAtT4) in both wild-type and *Zmrad17* meiocytes. The result displayed that nearly all of telomere signals were clustered and attached to the nuclear envelope in both wild-type (Figure 5A, *n* = 12) and *Zmrad17-1* (Figure 5B, *n* = 24) meiocytes at zygotene, indicating that *ZmRAD17* is not required for telomere bouquet formation.

The 5S ribosomal DNA (rDNA) is a tandemly repetitive sequence located on the long arm of chromosome 2 in maize and is often used to monitor homologous pairing (Li and Arumuganathan, 2001). To examine whether the disruption of *ZmRAD17* could impact the homologous chromosome pairing, FISH analysis using 5S rDNA as a probe was conducted. The results showed that only one 5S rDNA signal was constantly detected in both wild-type (Figure 5C, *n* = 23) and *Zmrad17-1* meiocytes (Figure 5D, *n* = 37) at pachytene, suggesting that *ZmRAD17* is not necessary for homologous pairing.

### *ZmRAD17* Is Indispensable for Synaptonemal Complex Assembly

**FIGURE 6:**
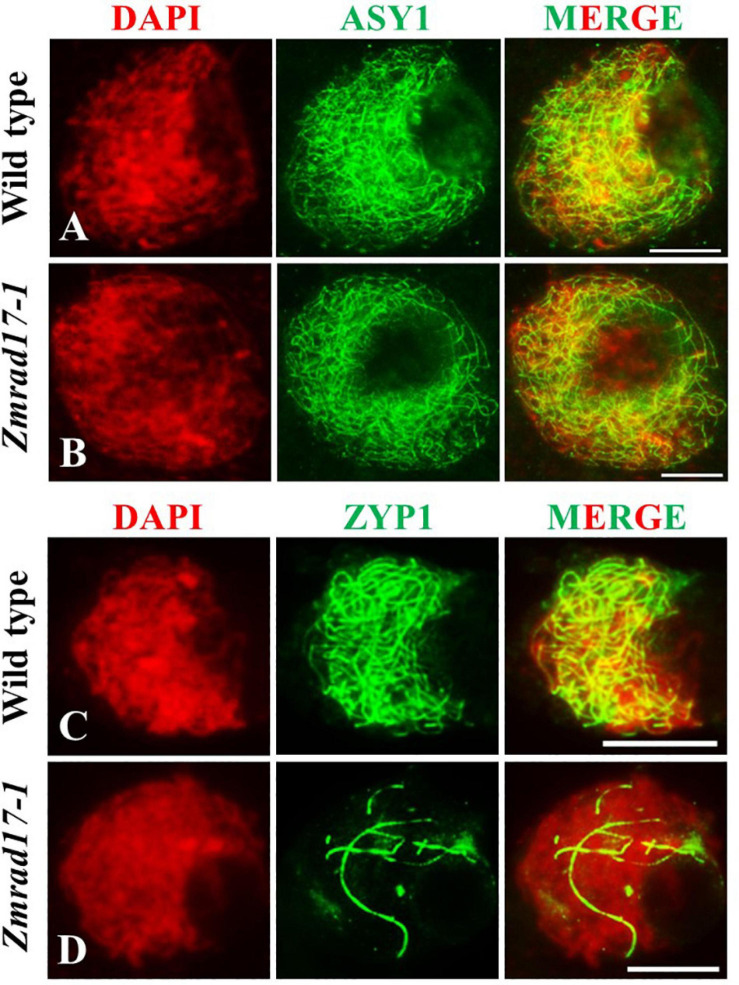
Immunolocalization of ASY1 and ZYP1 antibodies on meiotic chromosomes in wild type and Zmrad17-1 meiocytes. ASY1 **(A)** and ZYP1 **(C)** in wild type meiocytes; ASY1 **(B)** and ZYP1 **(D)** in Zmrad17-1 meiocytes. DAPI staining was used to indicate the chromosomes. Bars = 10 μm.

The synaptonemal complex (SC) is a protein scaffold linking homologous chromosomes to promote meiotic crossover formation (Cahoon and Hawley, 2016). To inspect the installation behavior of the SC, we conducted immunolocalization using antibodies against ASY1 and ZYP1 in both wild-type and *Zmrad17-1* meiocytes. ASY1, the axial element (AE) component of SC, localizes at chromosome axis (Sanchez-Moran et al., 2007; Sanchez-Moran et al., 2008). In the wild-type, ASY1 loading appeared as continuous linear signals along entire chromosomes at zygotene (Figure 6A, *n* = 10). Similar pattern of ASY1 distribution was observed in *Zmrad17-1* at the same stage (Figure 6B, *n* = 32), indicating that *ZmRAD17* is not required for AE installation.

ZYP1 constitutes the central element (CE) of SC (Higgins et al., 2005; Golubovskaya et al., 2011). At pachytene, ZYP1 signals in wild-type meiocytes formed continuous linear signals along the whole length of synapsed chromosomes (Figure 6C, *n* = 14). In contrast, although 18.2% of *Zmrad17-1* meiocytes showed a similar ZYP1 staining as wild-type, the remaining 81.8% of meiocytes exhibited short stretches of ZYP1 signals in *Zmrad17-1* (Figure 6D, *n* = 88). Taken together, these results indicate that *ZmRAD17* is indispensable for SC installation.

## Discussion

In yeast and mammals, it has been clarified that *RAD17* not only participates in mitosis, but also plays an important role in meiosis (Lydall et al., 1996; Grushcow et al., 1999; Shinohara et al., 2003; Budzowska et al., 2004). Deletion of *RAD24* in *S. cerevisiae* delayed DSB repair and resulted in abnormal recombination (Grushcow et al., 1999; Shinohara et al., 2003). In mouse, the mutation of *RAD17* caused embryonic lethality (Budzowska et al., 2004). In the model plant Arabidopsis, the defective *RAD17* was considered to have no strong effects on meiosis due to the normal fertility of both male and female, whereas the mutant displayed hypersensitive to DNA-damaging agents with the frequent presence of intrachromosomal HR during mitosis (Heitzeberg et al., 2004). In rice, the disruption of *RAD17* resulted in massive abnormal associations between non-homologous chromosomes, leading to enormous chromosome aggregations and fragments during male meiosis (Hu et al., 2018). In contrast, the loss-of-function of *RAD17* caused similar but much less severe effects on meiotic chromosome behaviors in maize, exemplified by subtle chromosome entanglement and fragmentation. Particularly, the unidirectional abnormality in male meiosis from the dysfunction of the maize *RAD17* seems strikingly different from rice, where both male and female were aborted (Hu et al., 2018). These findings highlight that although the participation of *RAD17* homologs in DSB repair is widely conserved, the precise effects of RAD17 on meiosis seem divergent among different organisms.

In budding yeast, the RAD24 (the homolog of RAD17) acts as the checkpoint clamp loader of the DNA damage response clamp 9-1-1 promoting assembly of synaptonemal complex and installation of ZMM proteins for CO formation (Shinohara et al., 2015; Crawford et al., 2018). In the *Zmrad17* mutant, the disturbed loading of ZYP1 protein supports the functional conservation of RAD17 in the SC installation between yeast and plant. In contrast, the SC formation seemed roughly normal in the *Osrad17* mutant, and the incomplete SC formation only occurred after combining *Osrad17* with mutation in ZMM proteins, such as ZIP4 or MSH5, implying that *OsRAD17* has to work cooperatively with ZMM proteins to promote homologous pairing and synapsis in rice (Hu et al., 2018). In this context, the redundancy between RAD17 and ZMM proteins in regulating the SC installation may not be critical in maize.

Chromosome fragmentation and entanglement are characteristic phenomena observed in mutants deficient in DSB repair machinery. Like the *Osrad17* mutants (Hu et al., 2018) and other related mutants such as *Zmcom1* (Wang et al., 2018), *Zmrad51c* (Jing et al., 2019), *Osxrcc3* (Zhang et al., 2015), *Atrad50* (Gallego et al., 2001; Bleuyard et al., 2004) and *Atmre11* (Samanic et al., 2013, 2016), the *Zmrad17* mutants showed the presence of chromosome fragmentation at prophase I. However, the severity of chromosome aberration seemed to be much less in *Zmrad17* when compared to the *Osrad17* mutants. A simple explanation for this discrepancy could be that other genes work redundantly with *ZmRAD17* in promoting accurate DSB repair. Alternatively, the other DSB repair pathway, such as classical non-homologous end-joining (C-NHEJ) (Shrivastav et al., 2008; Ceccaldi et al., 2016), which is routinely inhibited during meiotic DSB repair, could be active in the absence of the HR pathway (Hu et al., 2016). If this is true, such compensatory activity of C-NHEJ may vary between maize and rice. In this scenario, *ZmRAD17* might play a role in the DSB repair pathway choice, which has been suggested for the rice *OsRAD17* previously (Hu et al., 2018). Furthermore, as the CO formation appeared normal in the *Zmrad17* mutant, we propose that the repair of most DSBs by HR in *Zmrad17* is sufficient for the homologous recombination.

## Data Availability Statement

The original contributions presented in the study are included in the article/Supplementary Material, further inquiries can be directed to the corresponding author/s.

## Author Contributions

YH conceived and supervised the project. TZ, LL, and J-LJ conducted the experiments. TZ and YH prepared the manuscript. All authors read and approved the final manuscript.

## Conflict of Interest

The authors declare that the research was conducted in the absence of any commercial or financial relationships that could be construed as a potential conflict of interest.

## Footnotes

1 www.elabcaas.cn/memd/

2 https://maizegdb.org/
