# Supplementary figures and images for: ZmRAD17 Is Required for Accurate Double-Strand Break Repair During Maize Male Meiosis
Source: Front Plant Sci. 2021 Feb 26;12:626528. doi: 10.3389/fpls.2021.626528 (PMC7952653; doi:10.3389/fpls.2021.626528)

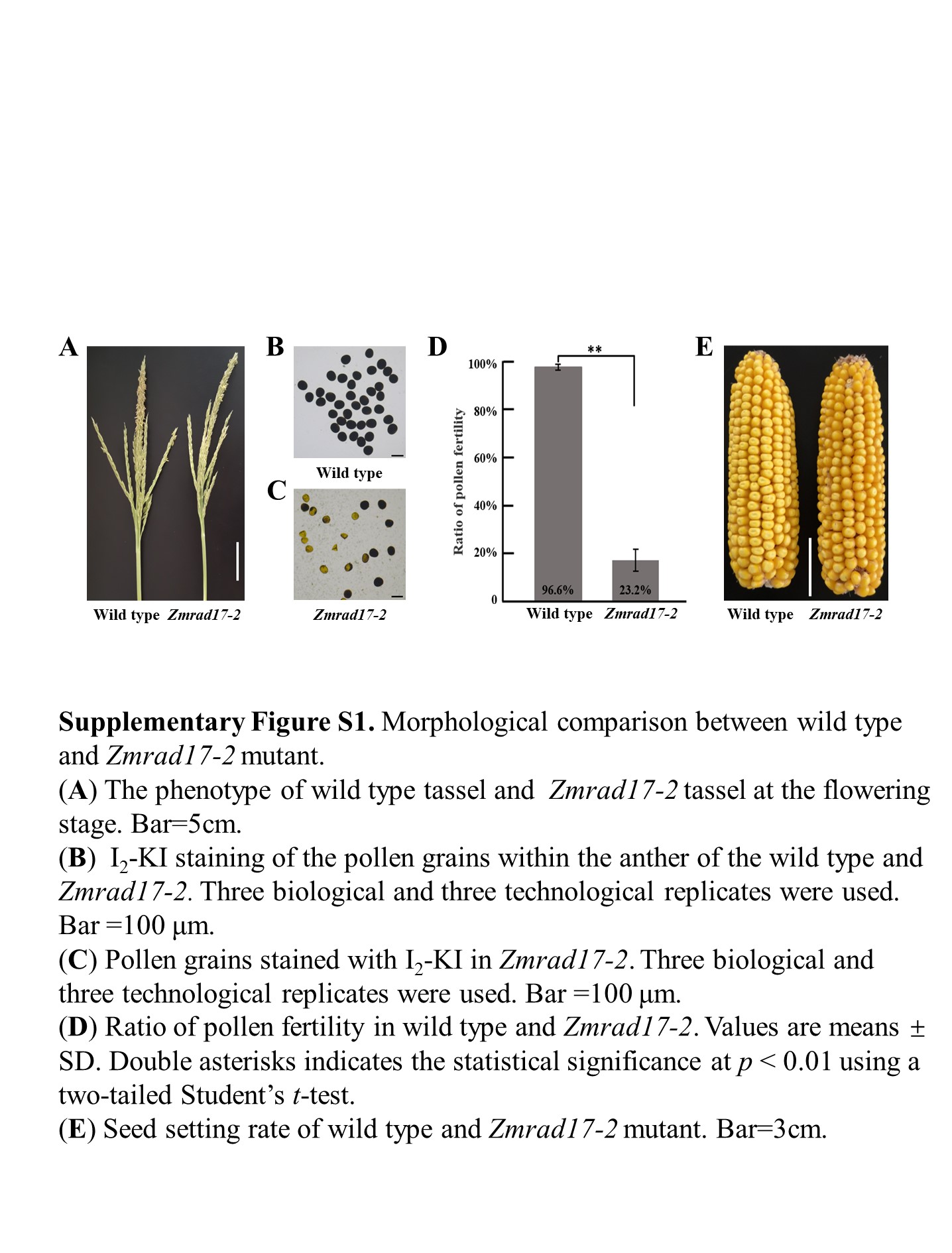

Supplement: Supplementary file 1 [file Image_1.JPEG]

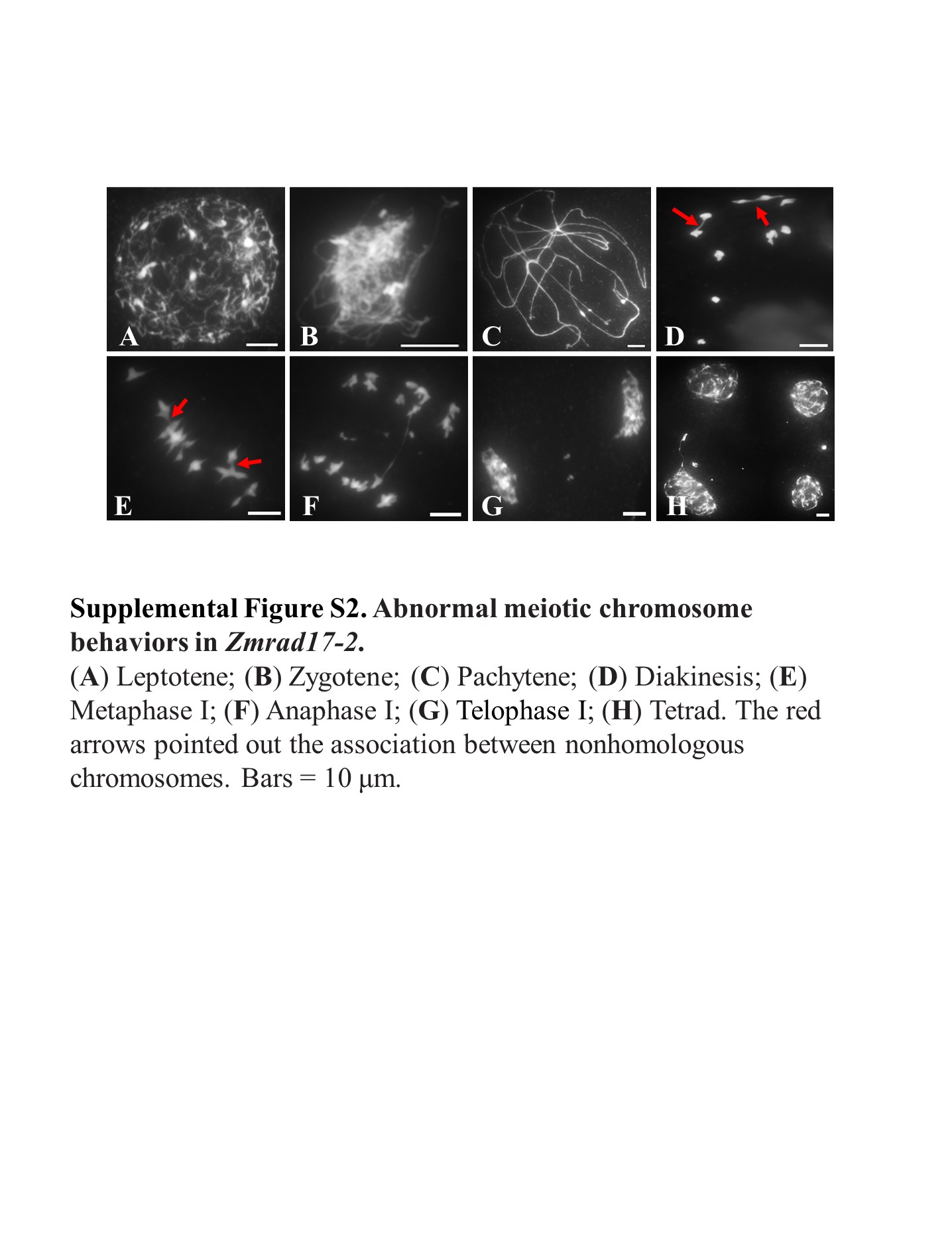

Supplement: Supplementary file 2 [file Image_2.JPEG]

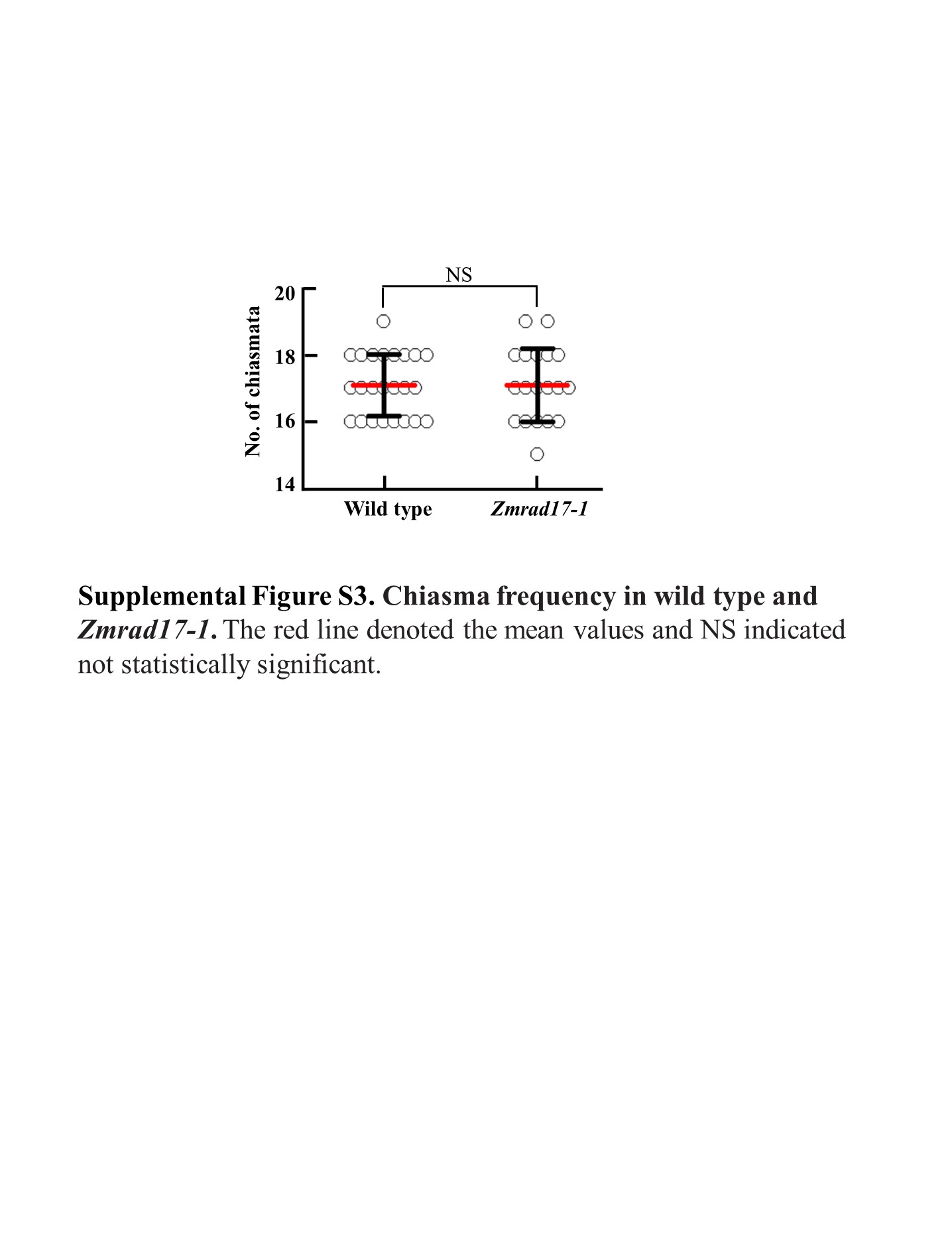

Supplement: Supplementary file 3 [file Image_3.JPEG]
