# Supplementary material for: ZmRAD17 Is Required for Accurate Double-Strand Break Repair During Maize Male Meiosis
Source: Front Plant Sci. 2021 Feb 26;12:626528. doi: 10.3389/fpls.2021.626528 (PMC7952653; doi:10.3389/fpls.2021.626528)
Supplement: Supplementary file 4 [file Data_Sheet_1.doc]

**Table S1.** List of primers used in this study.

| Name | Forward (5’-3’) | Reverse (5’-3’) |
| --- | --- | --- |
| **For genotyping** | | |
| Zmrad17-1 | GCTAGTCGTGCAGATTTCC | AGTGGTAGAAGACGAGAC |
| Zmrad17-2 | GAGAGTAACGATACTGCAAT | GTTGCCAATGCAGAACTATG |
| **For RT-qPCR**  ZmRAD17-RT  ZmUBQ1 | GAGAGTAACGATACTGCAAT TAAGCTGCCGATGTGCCTGCG | TAGAGGTGGCTATTTGATGC  CTGAAAGACAGAACATAATGAGCACA |
